# Test and Finite Element Analysis of a New Type of Double-Limb Double-Plate Connection Joint in Narrow Base Tower

**DOI:** 10.3390/ma14205936

**Published:** 2021-10-10

**Authors:** Hong Yan, Xianze Nie, Lei Zhang, Feng Yang, Mojia Huang, Tengfei Zhao

**Affiliations:** 1 Institute of Engineering Mechanics, Nanchang University, Nanchang 330031, China; yhtj@jju.edu.cn (H.Y.); mojiahuang@hotmail.com (M.H.); 2 College of Architectural Engineering and Planning, Jiujiang University, Jiujiang 332005, China; 6130085@jju.edu.cn; 3 China Nerin Engineering Co., Ltd., Nanchang 330031, China; 15907929824@163.com; 4 School of Civil and Architecture Engineering, East China University of Technology, Nanchang 330013, China; kittyyangfeng@126.com

**Keywords:** joint plate, carrying capacity, bending stress, numerical simulation, pipe double-limb double-plate joint

## Abstract

The connection between the leg members and diagonal members of the urban transmission line tower is mostly in the form of single-limb connection. This paper puts forward a new connection form of pipe double-limb double-plate connection joint, which is based on the model of key joints in an urban narrow base tower structure. The traditional pipe single-limb single-plate and new pipe double-limb double-plate joint are analyzed and studied from three aspects of theory, numerical simulation and experimental study. Through finite element analysis, it is obtained that the section stress of angle steel under eccentric load is 2.05 times of that under axial load, which is basically consistent with the 2.5 times of the theoretical calculation. This shows that the stress of the angle steel in the pipe double-limb double-plate joint is greatly reduced as the axial stress component, which can ensure the safety of the angle steel. Based on the theoretical analysis of the tensile force of two kinds of joints, through the test research and corresponding numerical simulation of pipe single-limb single-plate and pipe double-limb double-plate joints, under the same load, compared with pipe single-limb single-plate joints, the pipe double-limb double-plate joints designed in this paper can greatly reduce the stress of connection plates and members, and compared with the existing joint forms, the bending stress of joint plates can be reduced by about four times, which greatly improves the bearing capacity of the joint. The research on the pipe double-limb double-plate connection joint will provide the basis for the design of new connection joints of narrow base towers in urban areas.

## 1. Introduction

With the increasing tension of line corridors in suburban areas, narrow base steel tube towers are commonly used in transmission lines. The leg member of the narrow base steel pipe tower is steel pipe. The diagonal member is angle steel, and the connection form of the pipe single-limb single-plate joint is adopted between the diagonal member and the leg member [1,2,3]. Angle steel, as a diagonal member, has the advantages of a simple connection construction process and high efficiency, which is the premise for the widespread use of pipe single-leg single-plate joints. However, this connection method has the following disadvantages: The diagonal member is in an eccentric tension and compression state, prone to lateral instability, and the bending stress at the weld between the gusset plate and the leg member of steel pipe is large, which is unfavorable to the safety of the joint [4]. In order to address the problems of the pipe single-limb single-plate joint, we designed a new type of joint—pipe double-limb double-plate joint—which can effectively solve the problems caused by the pipe single-limb single-plate joint.

At present, domestic and foreign scholars mainly study the pipe single-limb single-plate joint from the aspect of the axial force. Cheng [5,6] analyzed the compression performance of the gusset plate with ANSYS, and the obtained elastic buckling load agrees well with the test value. It is noted that the elastic buckling load increases with the thickness of the gusset plate and the length of the diagonal brace member penetrating into the gusset plate. Professor Xingping Shu [7,8,9,10,11,12,13] found that the bearing capacity of the gusset plate decreases with the increase of the diameter thickness ratio of the main material. Professor Rui Cheng [14,15,16] found in the full-scale test of single-angle steel connection joints that the gusset plate is prone to out-of-plane instability during compression, and the eccentric stress of the gusset plate will greatly reduce its compression bearing capacity. Eccentricity only affects the yield bearing capacity of the gusset plate, but has little effect on its ultimate bearing capacity. Professor Yanzhong Ju [17,18,19] analyzed the failure mode of the gusset plate of a steel pipe angle steel composite tower. It is concluded that when the thickness ratio of the gusset plate to angle steel is greater than 1.2 under compression, angle steel will lose stability first. When the ratio is less than 1.2, the gusset plate is out-of-plane unstable, but the angle steel is unstable first.

Professor Guangzhao Li [20] obtained from the test of studying the failure mechanism of gusset plates that the failure forms of gusset plates can be divided into compression zone failure and tensile zone tear failure. Yong Guo and Bingnan Sun [21] obtained the calculation formula of stress concentration factor when studying the stress concentration of K-shaped steel pipe joints connected by gusset plates. Liqiang An and Dengjie Zhu [22] simulated the joint connection of a UHV steel pipe tower through the finite element method. The calculation results show that the influence of semi-rigid joint on the design results should be considered when calculating the internal force of a steel pipe tower. Jiabao Chen and Junfang Lei [23] found that the ultimate bearing capacity of K-joints is directly proportional to the thickness of the gusset plate and angle steel, but the length of the gusset plate and angle steel has little effect on it. Yongsheng Liu [24] monitored the influence on the mechanical performance and bearing capacity of K-shaped steel tubular joints by reducing the thickness of the gusset plate. Zhengliang Li and Hongjun Liu [25] verified the applicability of the calculation formula of the bearing capacity of the gusset plate with or without the stiffening ring through the full-scale test and finite element analysis of five groups of typical joints of the transmission tower.

Menghong Wang and Yingbo Bai [26], through the analysis of the bearing capacity of the joint under bending moment load, obtained that the bending bearing capacity of the joint is directly proportional to the diameter of the main pipe, but remains unchanged after reaching a certain degree, and the wall thickness of the main pipe has a great impact on the bearing capacity. Guohui Shen and Zhen Chen [27] concluded that contact plus preload is a relatively reasonable finite element simulation method to study the mechanical properties of bolted gusset plate shear connection. Bixiang Sun [28] obtained, through the full-scale test of single-angle steel joints, that the dangerous part of the gusset plate is the end area of the angle steel. Because the single-angle steel is subject to eccentric load, and the gusset plate may have torsional deformation while bending. Feng Li and Hongzhou Deng [29] concluded that the torsional bending instability of single-angle steel around the parallel axis is mainly controlled by the torsional bending instability in the study of the stability of angle steel members. Gao Qiang and Zhao Weijian [30] designed and manufactured 33 splice specimens with different parameters based on a new type of grouted sleeve and slotted rebar. Uniaxial tensile tests and a comparative analysis were carried out to study the influence of parameters, such as the inner cavity of sleeves, the anchorage length, and the grout strength, on the mechanical properties of grouted sleeve splices. P. Lehner [31] proposed a method to evaluate the residual life of existing steel structures in terms of fatigue damage. Zhijian Yang and Guochang Li [32] tested four prestressed high-strength concrete pile (PHC pile)–pile cap connections under low-cycle loading, and studied the test phenomena, failure modes, hysteretic performance, ductility, and bearing capacity. The PHC piles were reinforced with steel fiber and deformed bars and CFRP.

Most domestic research work considers the expansion of gusset plate under the axial stress state of single-angle steel, and does not consider the impact of eccentric stress of single-angle steel on gusset plate, the out-of-plane bending deformation of gusset plate during tension, and the failure mode of gusset plate under compression. These factors have a great influence on the strength of gusset plates. The pipe double-limb double-plate joint designed in this paper can avoid the above situation and enhance the stability and safety of the structure, which is of high practical significance.

Through the scale test and finite element analysis of pipe single-limb single-plate joint and pipe double-limb double-plate joint, this paper further studies the tensile performance of point plate when pipe double-limb double-plate joint is adopted, as well as the change of bending stress at the weld between gusset plate and leg member and the stress analysis of long and short angle steel. Mechanical characteristics of pipe single-limb single-plate joint and pipe double-limb double-plate joint are summarized. The advantages of pipe double-limb double-plate joint are verified by comparing test, finite element analysis, and theoretical analysis.

For convenience, the pipe single-limb single-plate joint is hereinafter referred to as PSLSPJ, and the pipe double-limb double-plate joint is hereinafter referred to as PDLDPJ.

## 2. Theoretical Analysis of Angle Steel Section Stress and Plate End Stress of PSLSPJ and PDLDPJ

### 2.1. Structural Forms of PSLSPJ and PDLDPJ

**Figure 1:**
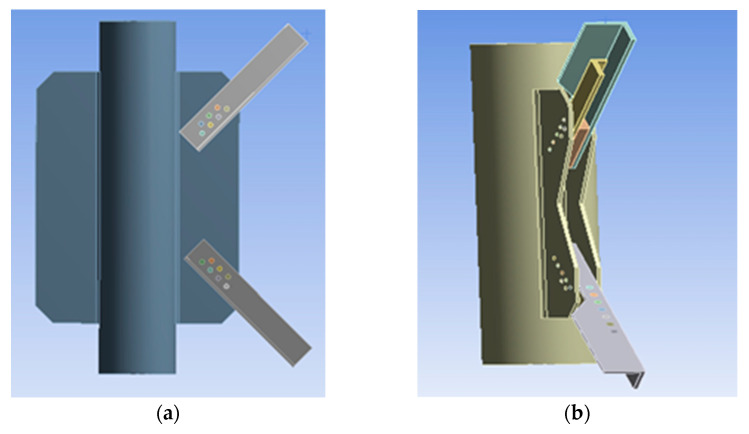
(**a**) Joint model of pipe single-limb single-plate; (**b**) joint model of pipe double-limb double-plate.

The PDLDPJ is improved from the PSLSPJ structure. The thickness of the gusset plate in the PDLDPJ is half of that in the PSLSPJ; two kinds of angle steel with the same specification and different lengths are used in the PDLDPJ, which are called long angle steel and short angle steel; A cushion block is padded between the long angle steel and the short angle steel, placed between the two gusset plates, and then connected with bolts, as shown in Figure 1.

### 2.2. Stress Analyses of the Angle Steel Cross Section under Axial Load and Eccentric Load Based on Theoretical Analysis

**Figure 2:**
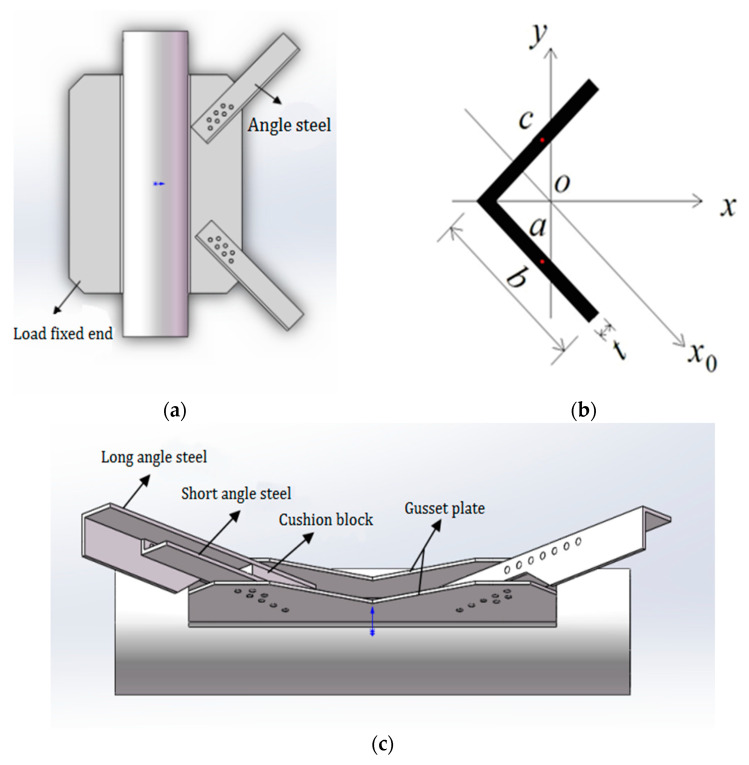
(**a**) Angle steel and its section characteristics of PSLSPJ; (**b**) calculation diagram of section characteristics of angle steel; (**c**) section characteristics of angle steel of PDLDPJ.

In the joint form of steel pipe leg member and angle steel diagonal member, when the angle steel in steel PSLSPJ is subjected to load, the angle steel is eccentric relative to the gusset plate, while the angle steel in steel PDLDPJ is connected with the gusset plate by the combination of two angles under load. Angle steel and gusset plate can be regarded as axial force. In the two joint forms, the section form of angle steel is shown in Figure 2, in which the limb width of angle steel is b and the limb thickness is t.

Ignore the influence of trimming and fillet of angle steel, it can be approximately considered that the area of angle steel is shown in Equation (1).

(1) A=2bt

The section moment of inertia of the x-axis is shown in Equation (2).

(2) $$I_{x}=\frac{1}{3}tb^{3}$$

Assuming that the axial force P is tensile force, it acts on point a of a single limb of angle steel. When calculating the stress at point a of angle steel, the axial force P shall be simplified to the centroid of angle steel for calculation. When simplifying, the action of bending moment shall be considered. The bending moment is shown in Equation (3).

(3) $$M=P\frac{b}{2}\times \frac{\sqrt{2}}{2}=\frac{\sqrt{2}}{4}Pb$$

The stress at point a of angle steel consists of two parts: One part is the stress generated by the direct action of the axial force is shown in Equation (4).

(4) $$\sigma_{a1}=\frac{P}{2bt}$$

The other part is the stress caused by bending moment is shown in Equation (5).

(5) $$\sigma_{a2}=\frac{M}{W_{x}}=\frac{3P}{4bt}$$

Thus the stress at point a is shown in Equation (6).

(6) $$\sigma_{a}=\sigma_{a1}+\sigma_{a2}=\frac{P}{2bt}+\frac{3P}{4bt}=\frac{5P}{4bt}=\frac{5}{2}\frac{P}{2bt}$$

When the axial force P acts on point O, the angle steel is subjected to axial force, and the stress at point O is shown in Equation (7).

(7) $$\sigma_{O}=\frac{P}{2bt}$$

According to Equation (6) and Equation (7), when the force acts at point a, the stress of angle steel under eccentric stress is 2.5 times that of the angle steel under axial stress at point O.

### 2.3. Comparison of the Gusset Plate End Stress between PSLSPJ and PDLDPJ Based on Theoretical Analysis

**Figure 3:**
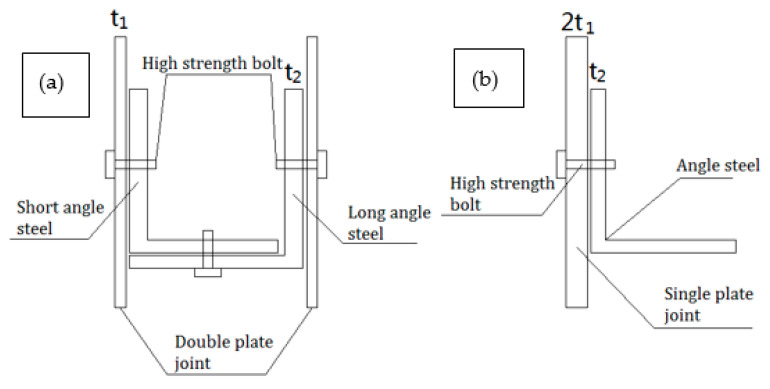
(**a**) Models of PDLDPJ; (**b**) models of PSLSPJ.

When the joint is connected by PSLSPJ, the load acts on the angle steel, which is eccentric for the gusset plate. The gusset plate will be subjected to the tensile and compressive load from the angle steel, and the bending moment caused by eccentricity will increase the stress at the plate end, which may lead to the damage of the gusset plate. Generally, the normal use of the member is ensured by increasing the thickness of the gusset plate. However, it will increase the weight of the overall structure and waste of materials. For the gusset plate of PDLDPJ, the connection with angle steel makes the gusset plate tend to bear axial force, which can greatly reduce the stress of the gusset plate. Connection form between the gusset plate and angle steel of the two joints is shown in Figure 3.

Assuming that the ratio of the force transmitted by the long and short angle steel in the PDLDPJ is 1:1, the load P/2 is applied to the gusset plate of the PDLDPJ on the long angle steel, and the limb width of the angle steel is L. At this time, the gusset plate is under axial stress, and then the end stress of the gusset plate is shown in Equation (8).

(8) $$\sigma_{1}=\frac{P}{2Lt_{1}}$$

For the gusset plate of PSLSPJ, the same load P is applied to the single-angle steel. At this time, angle steel is eccentrically stressed, resulting in additional eccentric bending moment to the gusset plate. The additional eccentric bending moment is shown in Equation (9). Thus, end stress of the gusset plate is shown in Equation (10).

(9) $$M=\frac{P(2t_{1}+t_{2})}{2}$$

(10) $$\sigma_{2}=\frac{P}{2Lt_{1}}+\frac{M}{\frac{4}{6}Lt_{1}^{2}}=\frac{8Pt_{1}+3Pt_{2}}{4Lt_{1}^{2}}$$

Then the ratio of plate end stress of the pipe single-limb single-plate gusset plate to the plate end stress of the pipe double-limb double-plate gusset plate is shown in Equation (11).

(11) $$\frac{\sigma_{2}}{\sigma_{1}}=\frac{\frac{8Pt_{1}+3Pt_{2}}{4Lt_{1}^{2}}}{\frac{P}{2Lt_{1}}}=4+\frac{3t_{2}}{2t_{1}}$$

It can be concluded from Equation (11) that, when the joint form adopts PSLSPJ, the plate end stress is at least four times that of PDLDPJ, and its ratio is related to the thickness $t_{2}$ of angle steel and the thickness $t_{1}$ of gusset plate.

## 3. Comparison of Finite Element Analysis Results and Theoretical Values of Angle Steel Section Stress and Plate End Stress of PSLSPJ and PDLDPJ

**Figure 4:**
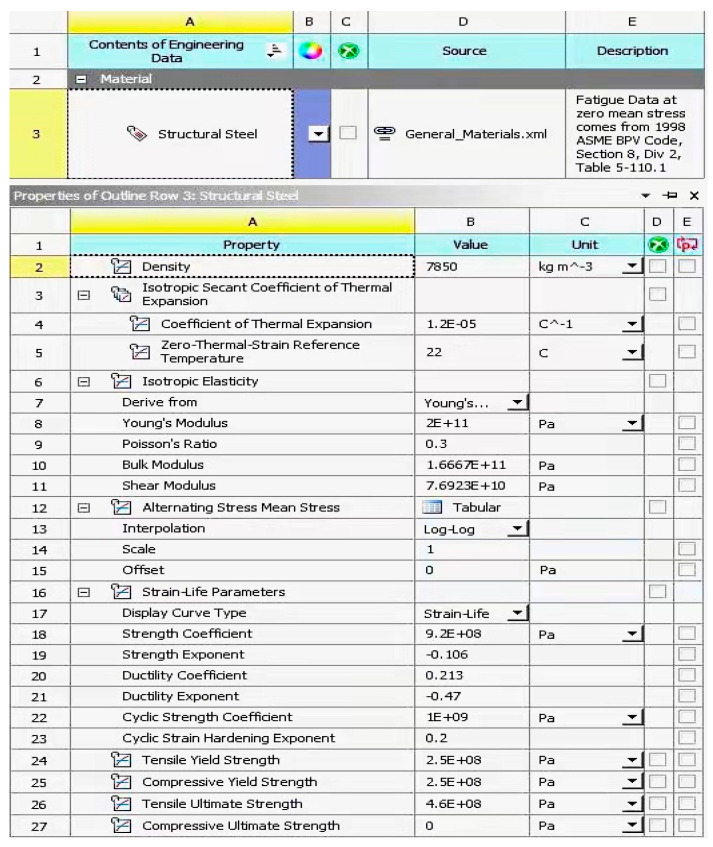
Material characteristics of the finite element model and test piece.

According to the previous theoretical analysis, it can be known that, compared with PDLDPJ, PSLSPJ has the disadvantages of large plate end stress, low stability, easy instability of angle steel, large bolt section stress, and small overall stiffness. For the plate end stress of the gusset plate, because the PSLSPJ is eccentrically stressed, the plate end of the gusset plate is not only subjected to tensile and compressive load, but also subjected to additional bending moment caused by angle steel eccentricity. The stress of PDLDPJ is axial force, which can avoid the generation of additional bending moment. For the angle steel in the joint, the angle steel of the PSLSPJ is eccentrically stressed, which is 2.5 times greater than the axial stress of the angle steel, and the angle steel of the PDLDPJ is axially stressed. In terms of overall stiffness, the PDLDPJ has relatively large overall stiffness, which can effectively prevent the occurrence of lateral instability. In terms of bearing capacity, the bearing capacity of PDLDPJ is stronger than that of PSLSPJ. In order to verify the accuracy of relevant theories, a finite element model consistent with the size of subsequent tests is established. Numerical analysis and verification are carried out in combination with the finite element analysis software ANSYS Workbench 17.0. The dimensional specifications of the finite element model are shown in Table 1 and the material characteristics of the finite element model are shown in Figure 4.

### 3.1. Ratio of Section Stress of Angle Steel under Eccentric Load and Axial Load Based on Finite Element Analysis

**Figure 5:**
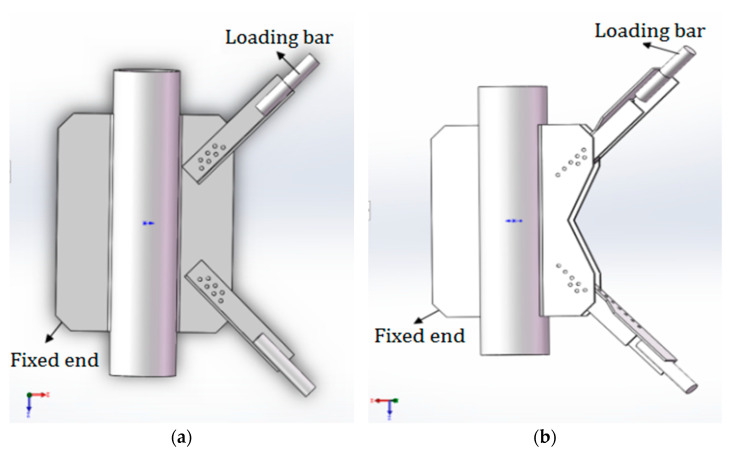
(**a**) Finite element model of PSLSPJ; (**b**) finite element model of PDLDPJ.

To verify the stress relationship of angle steel under eccentric load and axial load, finite element models of PSLSPJ and PDLDPJ were established, as shown in Figure 5.

Apply 60 kN eccentric force and 60 kN axial force to the angle steel in the PSLSPJ respectively and compare the section stress relationship of the angle steel in these two cases.

**Figure 6:**
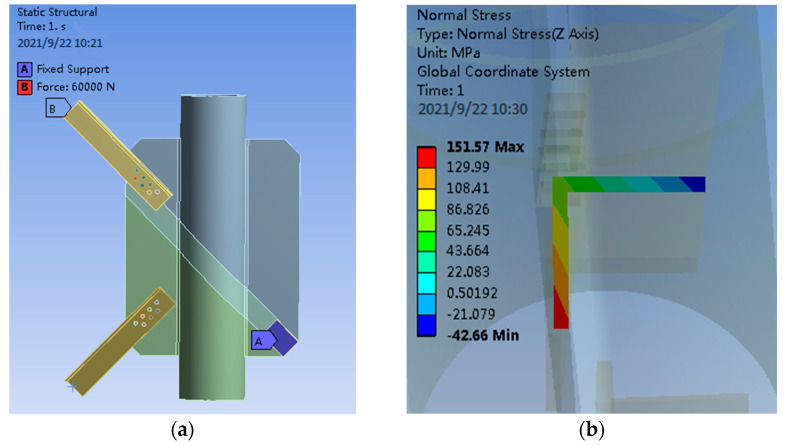
(**a**) Eccentric loading diagram of angle steel for PSLSPJ; (**b**) finite element results of angle steel for PSLSPJ.

- When the eccentric force is applied, a force of 60 kN is applied at 1/2 of one limb of the angle steel. The loading diagram and the stress results of finite element analysis are shown in Figure 6.

It can be seen from Figure 6 that, when the angle steel is eccentrically loaded, the maximum section stress on the angle steel is shown in the following Equation (12).

(12) $$\sigma_{1}=151.6\;\mathrm{MPa}$$

**Figure 7:**
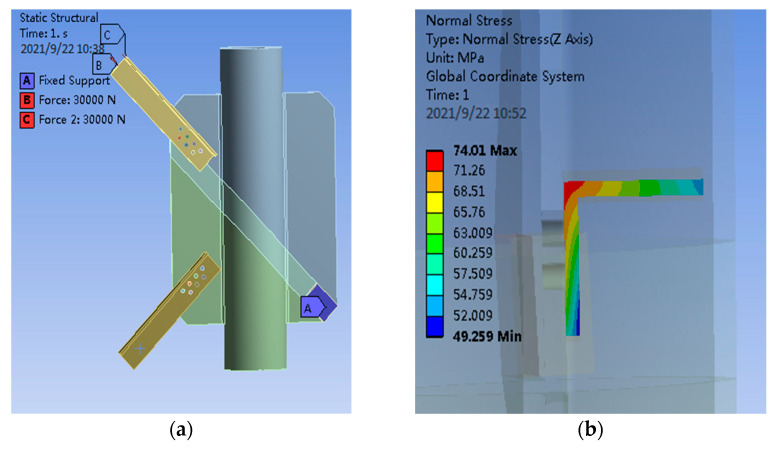
(**a**) Angle steel axle load diagram of PSLSPJ; (**b**) finite element results of PSLSPJ.

- When the axial load is applied, 30 kN tensile force is loaded at the midpoint of both limbs of the angle steel to obtain the load diagram and the section stress diagram of the angle steel, as shown in Figure 7.

It can be seen from Figure 7 that, when the axial load is loaded on the angle steel, the maximum section stress on the angle steel is shown in the following Equation (13).

(13) $$\sigma_{2}=74.01\;\mathrm{MPa}$$

According to the finite element results, the stress ratio of angle steel section under eccentric loading and axial loading is shown in the following Equation (14).

(14) $$\frac{\sigma_{1}}{\sigma_{2}}=\frac{151.60}{74.01}=2.05$$

Because real angle steel is trimmed and filleted, these factors are not considered in the establishment of a finite element model, thus the results obtained by the finite element method are different from the theoretical values. Ignoring these secondary factors, it can be considered that the section stress ratio of angle steel under eccentric load and axial load obtained by finite element analysis is 2.05 times, which is close to the ratio of 2.5 times obtained by theoretical derivation, indicating that the maximum stress difference between angle steel under eccentric load and axial load is 2.5 times.

**Figure 8:**
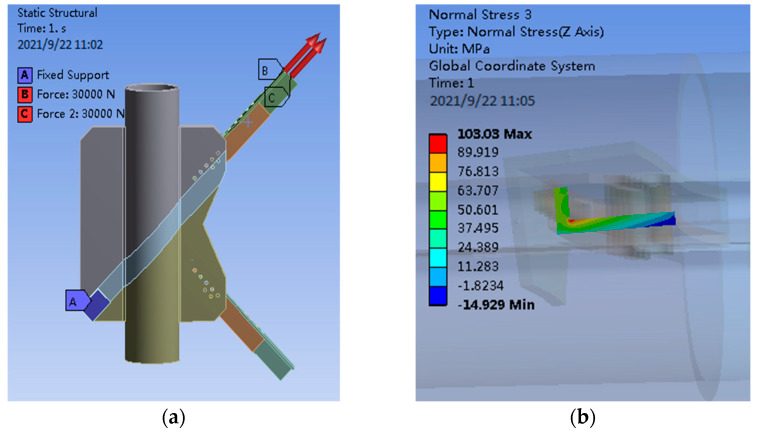

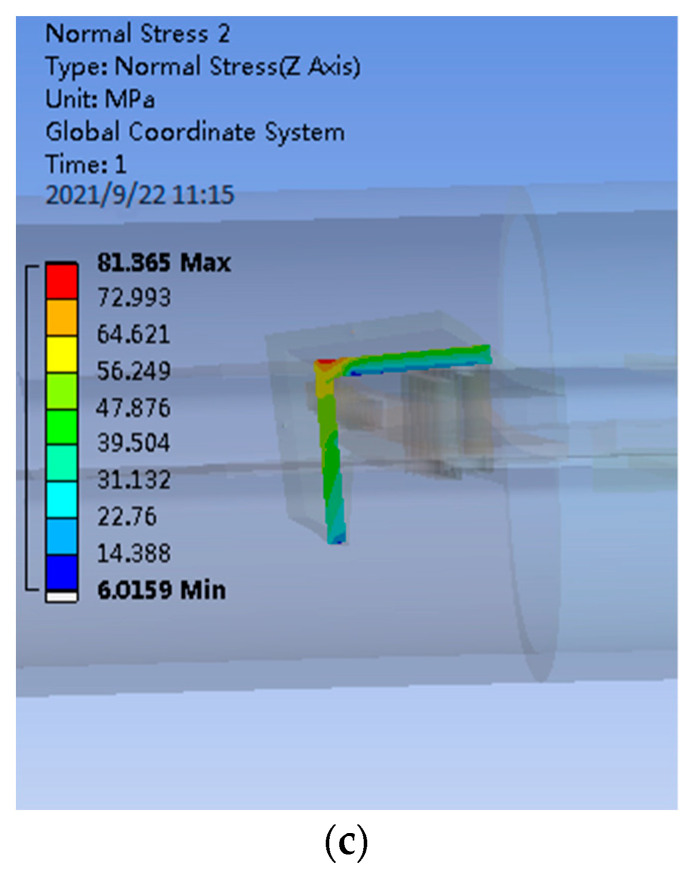
(**a**) Angle steel axle load diagram of PDLDPJ; (**b**) finite element results of short angle steel; (**c**) finite element results of long angle steel.

- The axial load is applied to the PDLDPJ. The loading and finite element analysis of the PDLDPJ are shown in Figure 8.

It can be seen from Figure 8, under the action of axial load, the average stress of short angle steel is 36.10 MPa and the average stress of long angle steel is 41.50 MPa in PDLDPJ. According to the finite element results of the section average stress of long and short angle steel, it can be obtained that the section average stress ratio of long and short angle steel is shown in Equation (15).

(15) $$\frac{\sigma_{\mathrm{Long}}}{\sigma_{\mathrm{Short}}}=\frac{41.50}{36.10}=1.10$$

Through the finite element calculation, the PDLDPJ is subjected to an external force; the section stress ratio of long and short angle steel is 1.1 times. It is consistent with the assumption that the load is evenly distributed on the long and short angle steel in the previous theoretical analysis.

### 3.2. Comparison of Plate End Stress between PSLSPJ and PDLDPJ Based on Finite Element Analysis

**Figure 9:**
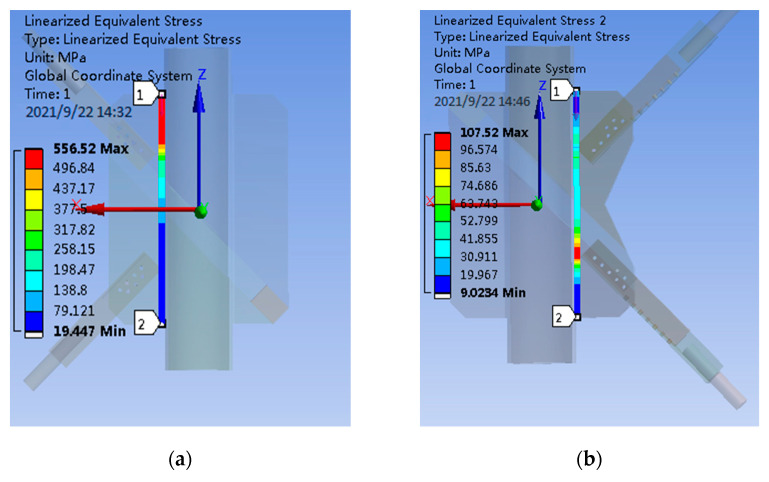
(**a**) End stress of plate of PSLSPJ; (**b**) end stress of plate of PDLDPJ.

The finite element model is established according to the specimen size, loading, and restraint mode used for subsequent tests, the model is loaded, the path along the weld direction is established on the gusset plate, and the equivalent stress on the path is extracted. As shown in Figure 9, the plate end stress of PSLSPJ and PDLDPJ is compared to verify the relationship with the theoretical solution.

It can be seen from Figure 9, according to the path equivalent stress diagram on the gusset plate of PSLSPJ, the maximum stress is shown in the following Equation (16).

(16) $$\sigma_{\mathrm{single}}=556.50\;\mathrm{MPa}$$

The maximum stress at the plate end of PDLDPJ is shown in the following Equation (17).

(17) $$\sigma_{\mathrm{double}}=107.50\;\mathrm{MPa}$$

Then the ratio of nodal plate end stress of PSLSPJ to PDLDPJ is shown in Equation (18).

(18) $$\frac{\sigma_{\mathrm{single}}}{\sigma_{\mathrm{double}}}=\frac{556.50}{107.50}=5.20$$

According to the theoretical Formula (6) derived above, the ratio of nodal plate end stress of PSLSPJ and PDLDPJ is shown in Equation (19).

(19) $$\frac{\sigma_{\mathrm{single}}}{\sigma_{\mathrm{double}}}=\frac{\frac{8Pt_{1}+3Pt_{2}}{4Lt_{1}^{2}}}{\frac{P}{2Lt_{1}}}=4+\frac{3t_{2}}{2t_{1}}=4+\frac{3}{2}\times \frac{5}{4}=5.80$$

According to the stress ratio of the gusset plate end of the PSLSPJ and the PDLDPJ calculated by the finite element analysis and theoretical calculation, it can be seen that it is basically consistent with the results of the plate end stress relationship deduced by the theory. Plate end stress of PSLSPJ is four times higher than that of PDLDPJ, and the multiple is related to the ratio of angle steel and gusset plate thickness.

In short, through the finite element analysis of the stress relationship of the angle steel of the PSLSPJ and the PDLDPJ under eccentric and axial loads and the stress relationship at the end of the gusset plate, the relevant theoretical analysis expressions are verified. The following conclusions are obtained.

- Through finite element analysis, it is obtained that the section stress of angle steel under eccentric load is 2.05 times of that under axial load, which is basically consistent with the 2.5 times of theoretical calculation. Therefore, it shows that the stress of the angle steel in the PDLDPJ is greatly reduced, which can ensure the safety of the angle steel.
- The finite element analysis shows that, under the same load, plate end stress of PSLSPJ is 5.2 times of that of PDLDPJ, which is basically consistent with the 5.8 times deduced from the theory. The plate end stress is related to the thickness of angle steel and gusset plate. At the same time, it can be seen that the PDLDPJ can effectively reduce the end stress of the gusset plate.

## 4. Static Load Test and Finite Element Comparative Analysis of PSLSPJ and PDLDPJ

### 4.1. Static Load Tests of PSLSPJ and PDLDPJ

#### 4.1.1. Purpose of Static Load Test

There is no corresponding joint design method for PDLDPJ in the current code. Considering the large ultimate load borne by diagonal members, it is very necessary to carry out static load test on key joints to verify the ultimate bearing capacity of PDLDPJ. The purpose of the test is the following:

- Through the static load test, the force transmission path, failure mechanism, and ultimate bearing capacity of pipe limb double-plate joint is obtained.
- Through the static load test, the ultimate bearing capacity of PSLSPJ is given and compared with that of the PDLDPJ.
- Comparing the measured results with the finite element analysis results, this paper analyzes the advantages of PDLDPJ, provides theoretical and experimental support for the design and installation of PDLDPJ, and provides strong technical support for the engineering application of PDLDPJ.

#### 4.1.2. Design of the Test Device and Test Piece

**Figure 10:**
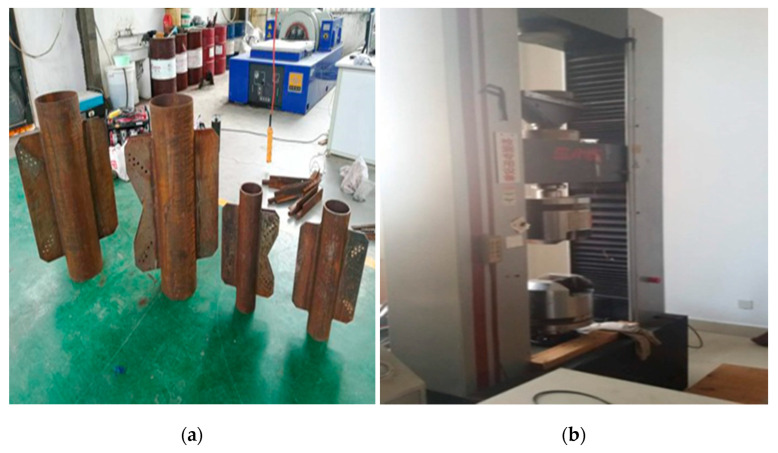
(**a**) Physical drawing of specimen; (**b**) electro-hydraulic servo universal testing machine.

The test is carried out on the electro-hydraulic servo universal testing machine. As shown in Figure 10. The testing machine is a double space structure, the tension space is between the upper and lower beams, and the compression space is between the lower beams and the test bench. The conversion of tension and compression test is convenient. The load under tension and compression conditions can reach 600 kN, meeting the space requirements of the test.

In this test model, the top limit load condition of a narrow base transmission tower in an urban area is taken as the design load, and two scale models are designed (the scale is about 1:5). Among them, the gusset plate on the other side of the leg member that is not connected with the angle steel is designed to facilitate fixation during the test, but not in the actual situation. The size and relevant parameters of the test piece are shown in Table 1, and the real object of the test piece is shown in Figure 10.

#### 4.1.3. Loading Principle

The loading method adopts step-by-step loading. The load of each level is 10 kN, and finally loaded to the design load. The axial force is loaded by the universal testing machine. The gusset plate on one side of the test piece is fixed by the lower fixture, and the loading bar is clamped by the upper fixture and applied with tension. During loading, the lower clamp remains fixed and the upper clamp is stretched in the vertical direction. The three-dimensional model of specimen loading is illustrated in Figure 5.

#### 4.1.4. Test Scheme

**Figure 11:**
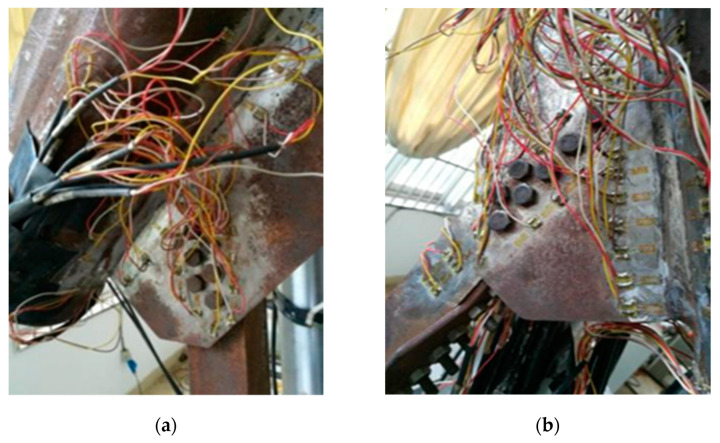
(**a**) Diagram of strain gauge arrangement of PSLSPJ; (**b**) diagram of strain gauge arrangement of PSLSPJ.

**Figure 12:**
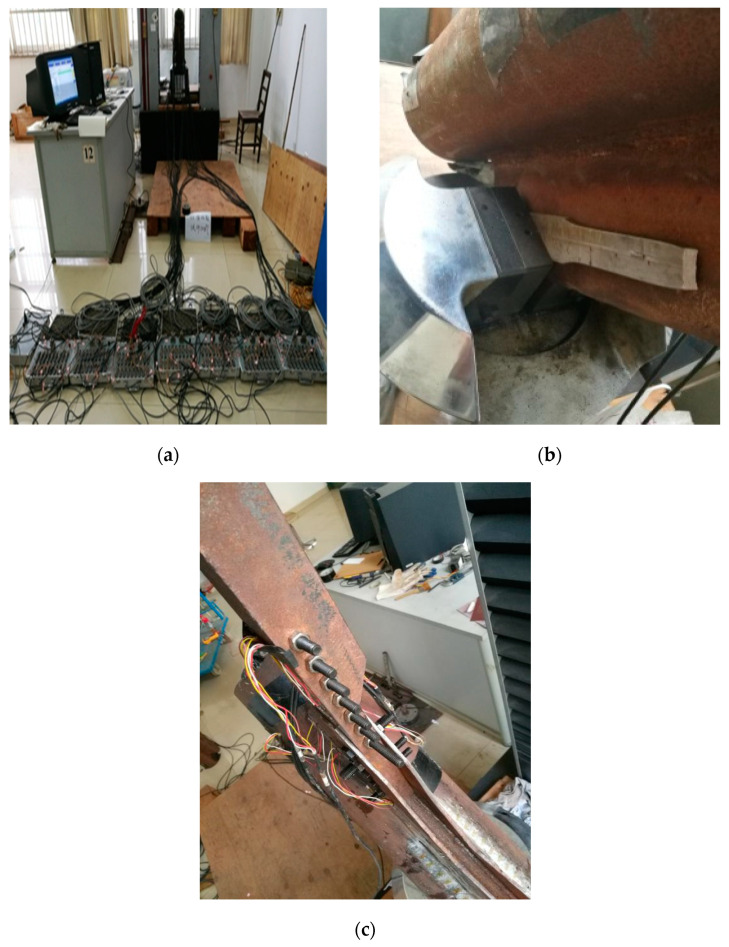
(**a**) Test loading device and strain acquisition system; (**b**) PSLSPJ installed on testing machine; (**c**) PDLDPJ installed on testing machine.

Test content: In order to track the stress on the welds of the gusset plate and the angle steel, unidirectional strain gauges are arranged at the corresponding positions, with a total of 90 measuring points, including 36 PSLSPJs and 44 PDLDPJs. The specific layout plan of the strain gauges on the front of the joint is shown in Figure 11, in which the front and back sides of the strain gauges are symmetrically arranged. The leg member measuring points is located next to the connecting weld between the gusset plate and the leg member and is distributed along the axial direction. The gusset plate is mainly arranged along the weld and around the bolt. The strain gauges on the angle steel are arranged along the action direction of the force. The test loading device and strain acquisition system are illustrated in Figure 12.

#### 4.1.5. Static Load Test Procedure

During the static load test of PSLSPJ and PDLDPJ, the test preparation and loading shall be carried out in accordance with the test steps, generally in accordance with the following steps:

- Label each test piece. According to the proposed measuring point position, after grinding, complete the pasting of the strain gauge and mark the strain gauge.
- Install the experimental loading tooling, install the test piece, and conduct preliminary positioning to make sure that the test piece is in the vertical state. The base of the test piece at the tube sheet joint is clamped and connected with the base loading device, so that the test piece is in the axial stress state during loading.
- Before the formal loading of the test piece, carry out preloading and check whether the strain gauge and strain collector work normally.
- When the specimen is formally loaded, it shall be loaded according to the prepared loading scheme, and the load shall be maintained for 30 s after each level of loading, so as to facilitate the collection of stable data, and then the next step of loading.
- The points of the specimen with large strain are mainly concentrated in the distribution of the leg member along the weld, the distribution area along the weld on the gusset plate, and the side of the long and short angle steel on the PDLDPJ. This part should be observed in the test.
- Complete the test under the pull-out condition of tube sheet joints. The collected data are processed and compared with the finite element results and theoretical values.

### 4.2. Finite Element Analyses of PSLSPJ and PDLDPJ

#### 4.2.1. Finite Element Analysis of Gusset Plate, Bolt, and Angle Steel in PSLSPJ

**Figure 13:**
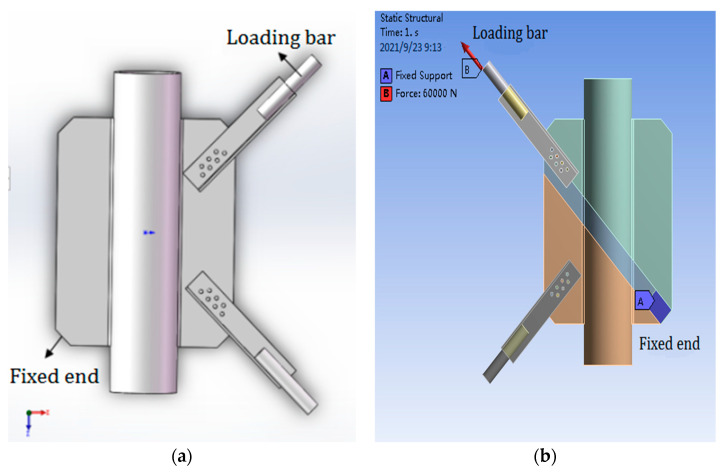

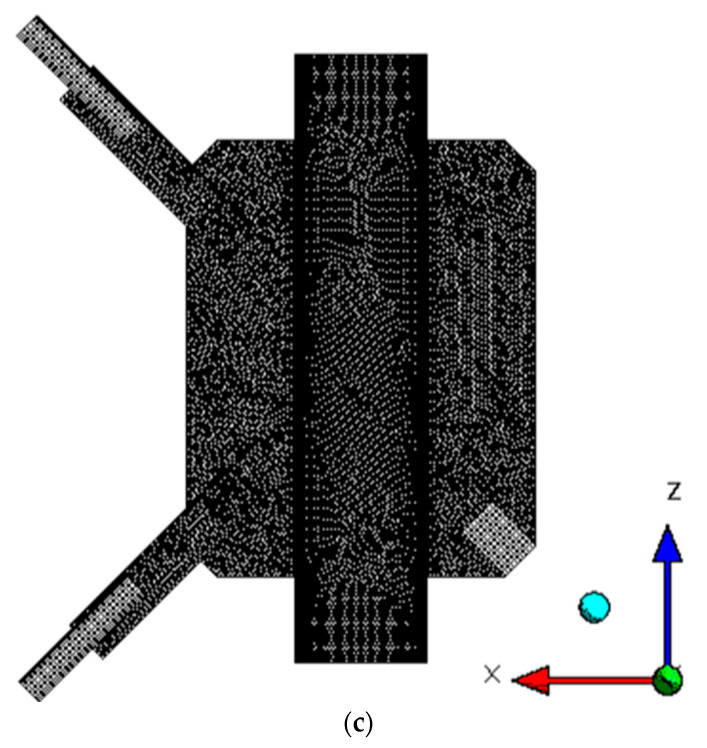
(**a**) Finite element model of PSLSPJ; (**b**) loading diagram of PSLSPJ; (**c**) meshing diagram of PSLSPJ.

As shown in Figure 13, according to the size of the PSLSPJ specimen used in the previous test, the solid model diagram is established by SOLIDWORKS 2019 and the finite element model diagram is imported into ANSYS Workbench, in which end A represents the fixed end and end B represents the loading rod, that is, the loading end. The software used in the finite element calculation is ANSYS Workbench 17.0. The method of generating mesh by the software can be set to generate mesh automatically and the element size is 2.5 mm. The meshing diagram is shown in Figure 13c.

**Figure 14:**
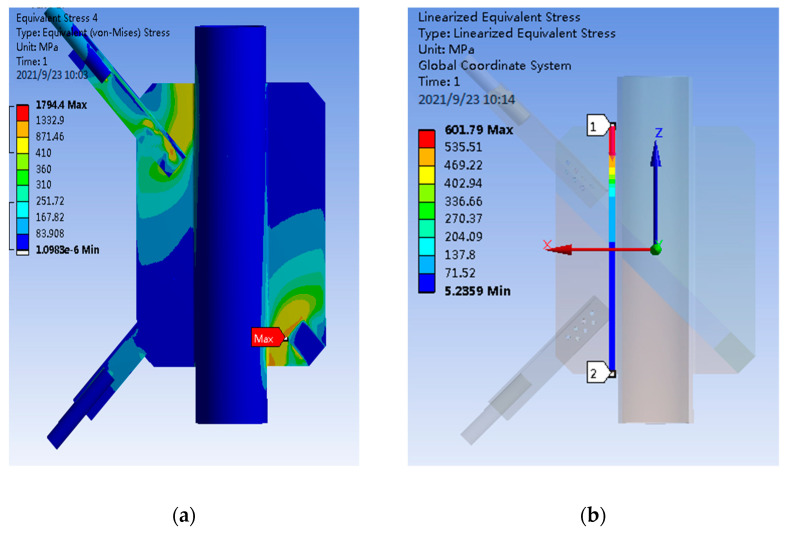

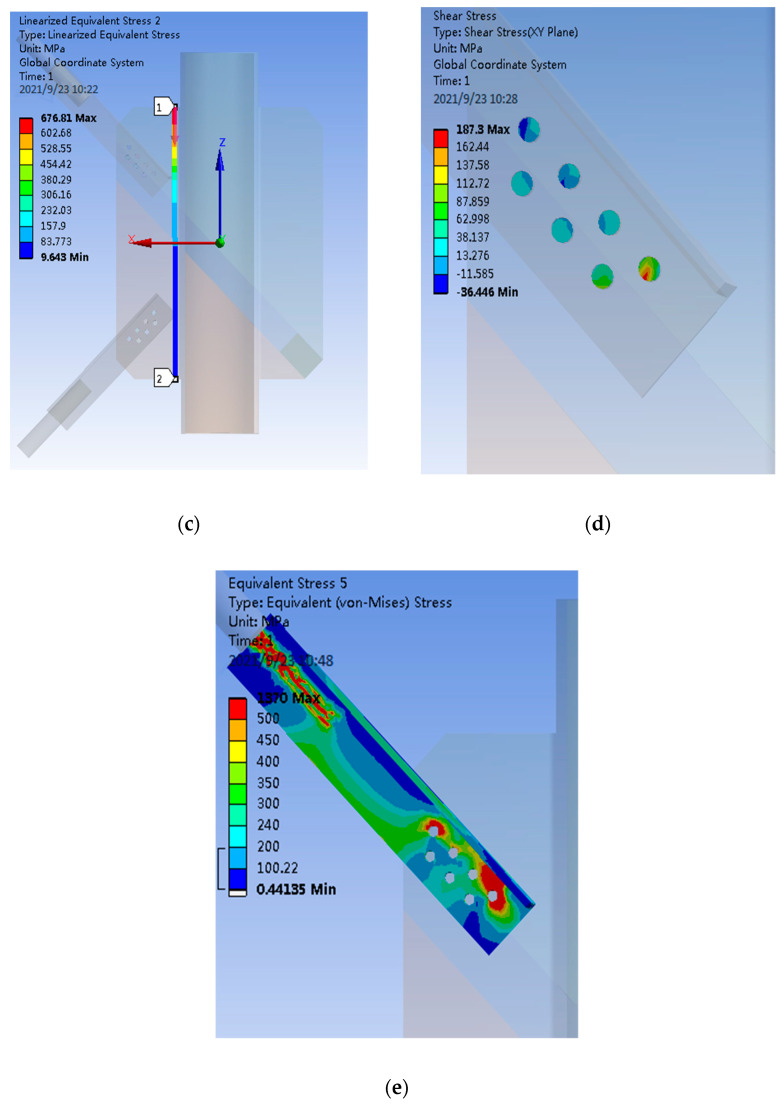
(**a**) Overall stress distribution of PSLSPJ; (**b**) stress distribution of the first path in the weld direction; (**c**) stress distribution of the second path in the weld direction; (**d**) stress distribution of connecting bolt of PSLSPJ; (**e**) stress distribution of angle steel of PSLSPJ.

During the finite element calculation of PSLSPJ, the load is the same as the test design value, which is 60 kN uplift force. According to the nephogram of joint stress obtained by the finite element calculation, the stress diagram on the path corresponding to the strain gauge was pasted in the test, in addition to the stress diagram of the connecting bolt and angle steel, as shown in Figure 14.

**Figure 15:**
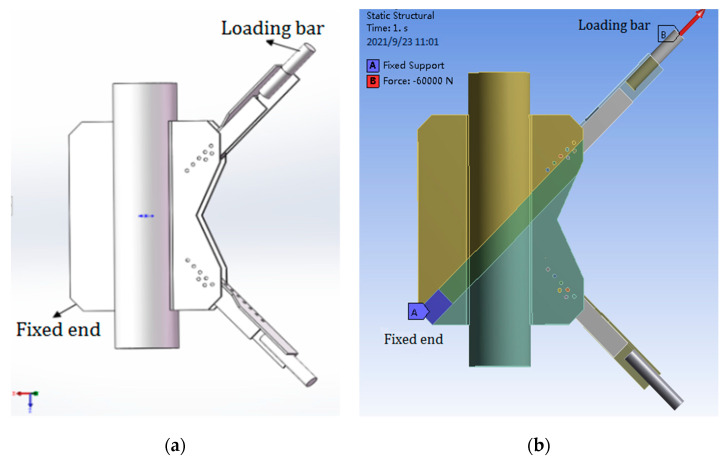

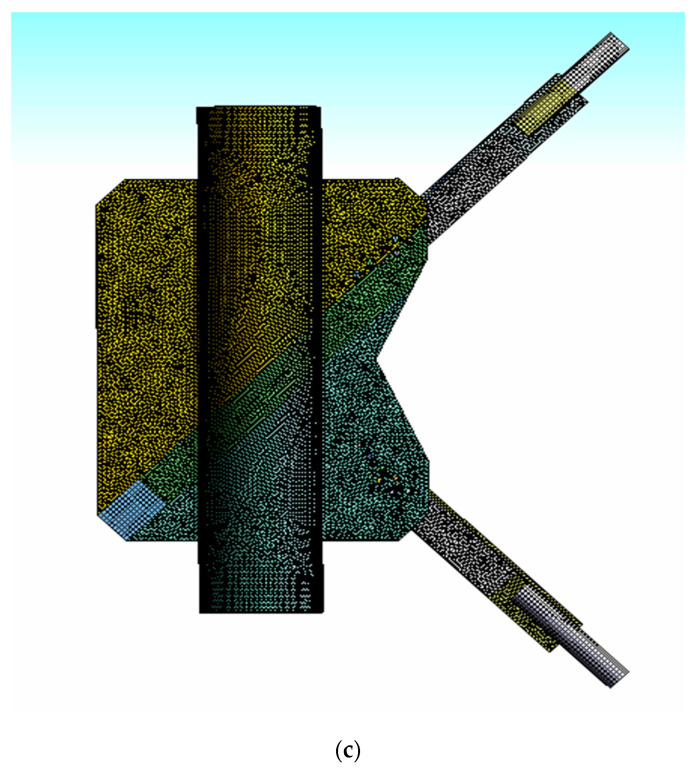
(**a**) Finite element model of PDLDPJ; (**b**) loading diagram of PDLDPJ; (**c**) meshing diagram of PDLDPJ.

From the stress nephogram in Figure 15 of PSLSPJ, it can be concluded that:

- The large stress area of the whole model is located at the connection between the angle steel and gusset plate and at the fixed end. The stress at the fixed end can be ignored. The stress in the connection area between the angle steel and the gusset plate is large, and local damage may occur.
- The stress results of the two paths taken along the weld direction on the gusset plate show that the maximum stress appears at the plate end of the gusset plate, and the maximum stress is about 600 MPa, and the farther away from the plate end, the smaller the stress.
- The stress distribution of connecting bolts shows that the stress of two bolts of the bolt group is large, the maximum stress is 187 MPa, and the stress of the middle bolt is small.
- Inclined stress of the angle steel of the pipe single-leg single-plate joint is large, and the stress in most areas is about 300 MPa. The stress at the edge of the angle steel near the gusset plate is greater than that of other parts, and the stress is between 300 to 350 MPa.

#### 4.2.2. Finite Element Analysis of Gusset Plate, Bolt, and Long and Short Angle Steel in PDLDPJ

**Figure 16:**
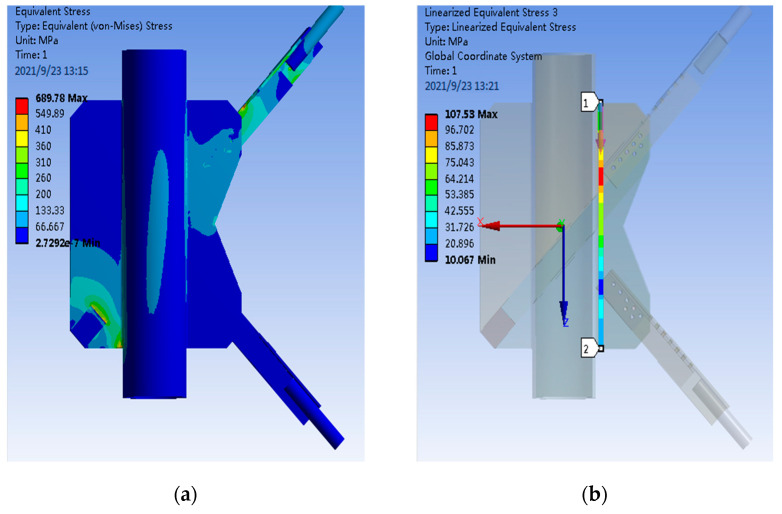

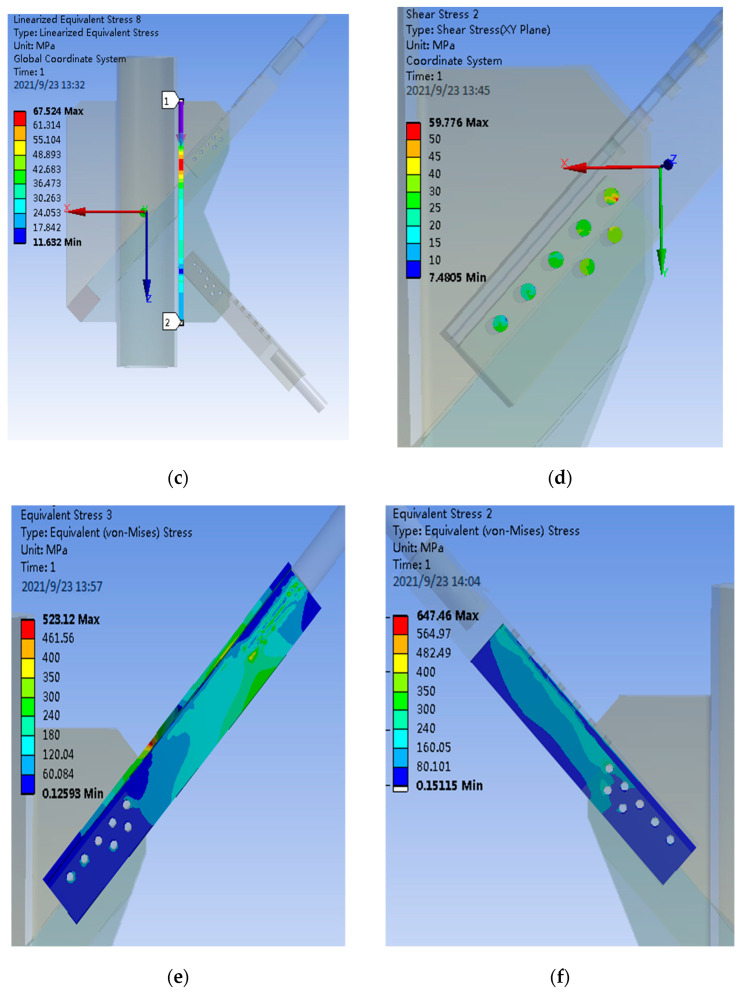
(**a**) Overall stress distribution of PDLDPJ; (**b**) stress distribution of the first path in the weld direction; (**c**) stress distribution of the second path in the weld direction; (**d**) stress distribution of connecting bolt of PDLDPJ; (**e**) stress distribution of long angle steel of PDLDPJ; (**f**) stress distribution of short angle steel of PDLDPJ.

As shown in Figure 15, according to the size of the PDLDPJ specimen used in the previous test, the solid model diagram shown in Figure 16 and the finite element model diagram imported into ANSYS Workbench are established by SOLIDWORKS, in which end A represents the fixed end and end B represents the loading rod, that is, the loading end. The software used in the finite element calculation is ANSYS Workbench 17.0. The method of generating mesh by the software can be set to generate mesh automatically. And the element size is 2.5mm. The meshing diagram is shown in Figure 15c.

After the grid division and the same loading and restraint as the test, the calculated overall stress of the PDLDPJ, the stress along the weld direction on the joint plate, the section stress of the connecting bolt and the stress distribution of the long and short angle steel are shown in Figure 16.

According to the stress distribution obtained by finite element calculation, as shown in Figure 16, it can be concluded that:

- The overall stress of PDLDPJ is less than 130 MPa in most areas, which is far less than the yield stress of the material.
- For the stress on the two paths along the weld direction on the gusset plate, the maximum stress on the gusset plate on one side of the short angle steel is 107 MPa, and the maximum stress on the other side is 67 MPa.
- The stress distribution of connecting bolts is the same as that of the PSLSPJ. The stress at both ends of the bolt group is large, the middle is small, and the maximum stress is 60 MPa.
- The maximum stress of the long angle steel in the PDLDPJ is 520 MPa, which occurs at the variable section of one leg of the angle steel, with stress concentration. Most of the stresses in other areas are within 240 MPa, meeting the yield stress requirements. The maximum stress on the short angle steel is 647 MPa, but it occurs at the place in contact with the bolt, which belongs to the contact stress and does not affect the normal use of its components. The stress in other areas is less than 300 MPa, meeting the requirements.

### 4.3. Comparative Analyses of Bearing Capacity Test Results and Finite Element Results of PSLSPJ and PDLDPJ

#### 4.3.1. Comparative Analysis of Bearing Capacity Test Results and Finite Element Results of PSLSPJ

During the bearing capacity test of the test piece of the PSLSPJ, a steel block with a thickness of 20 mm is padded on the side of the lower fixed end of the PSLSPJ to prevent the test piece from large eccentricity and additional eccentric bending moment.

According to the test data, the bearing capacity of the PSLSPJ is 131 kN, which complies with the requirements of the design load. After the specimen is removed from the test machine, it is found that the angle steel and connecting bolts are damaged.

In the test of PSLSPJ, the data collected by the strain collector are converted into stress and finite element calculation data, as shown in Table 2. The strain gauge numbered 1–9 are located on the front of the PSLSPJ, and the strain gauge numbered 19–27 are located on the back of the PSLSPJ.

**Figure 17:**
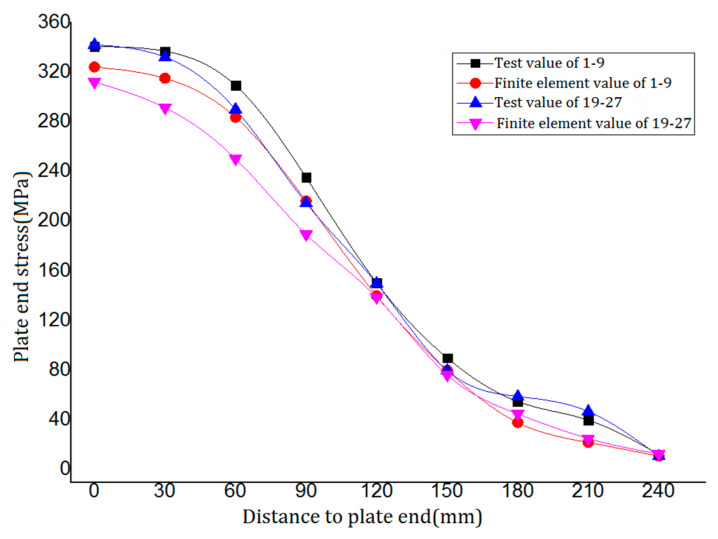
Comparison between finite element value and test value of plate end stress of PSLSPJ.

According to the data in Table 2, the relationship between the test value along the weld direction and the finite element value on the gusset plate of PSLSPJ is obtained, as shown in Figure 17.

As can be observed in Figure 17, the stress of the gusset plate along the weld direction decreases with the increase of the distance from the plate end, and the decreasing trend near the angle steel is more obvious. Experimental values of tube single-limb single-plate joint are in good agreement with the finite element values, and the change trend is basically the same, so the previous assumptions are verified to be reasonable.

According to the test and finite element analysis, the maximum bending stress at the end of the gusset plate is 340 MPa, and the structure is still in the elastic stage, which meets the design requirements.

#### 4.3.2. Comparative Analysis of Bearing Capacity Test Results and Finite Element Results of PDLDPJ

Through the bearing capacity test of PDLDPJ specimen, it can be obtained that the bearing capacity of PDLDPJ is 165.7 kN, which definitely meets the requirements of the design load (the design load is required to be 60.6 kN). After the specimen is removed from the testing machine, it is found that the angle steel and connecting bolt are damaged.

In the test of PDLDPJ, the data collected by the strain collector are converted into stress and the data obtained by finite element calculation are shown in Table 3. At the later stage of loading, only the strain gauge numbered 1–4 are located on the front side of the PDLDPJ (short angle steel side) and the strain gauge numbered 23–26 are located on the back side of the PDLDPJ (long angle steel side) to work normally. Therefore, Table 3 only lists the stress comparison of these points.

**Figure 18:**
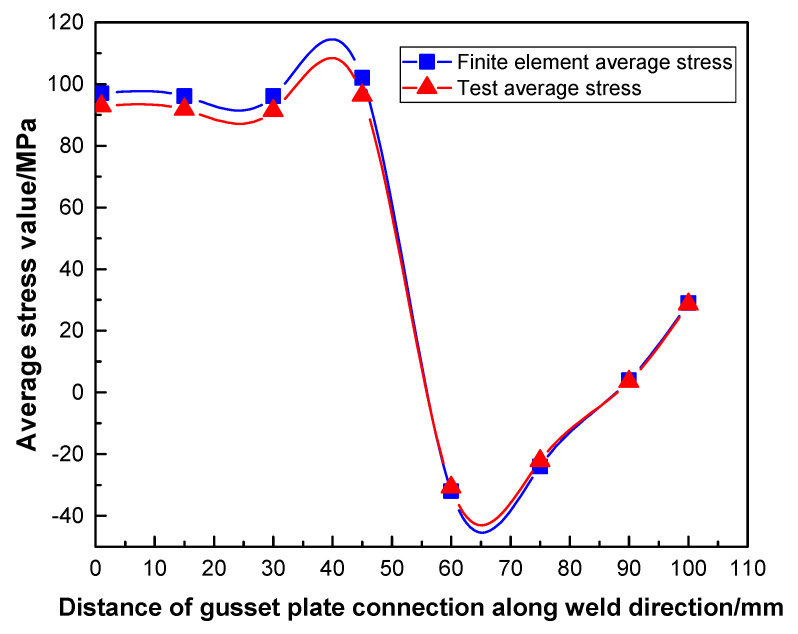
Analysis of finite element and experimental values of PDLDPJ.

According to Table 3, the average stress of long and short angle steel along the weld direction on the joint plate of PDLDPJ can be obtained in the test and finite element. Numbers 1–4 and 23–26 are the stress values on the front and back of the weld, respectively. Take the flat value of the stress on the front and back to obtain the comparison results of the test and finite element, as shown in Figure 18.

It can be seen from Figure 18 that the test value of PDLDPJ is in good agreement with the finite element value and the change trend is basically the same. It can be obtained that the stress distribution on long and short angle steel meets the average distribution. Therefore, the previous hypothesis is verified to be reasonable.

According to the test and finite element analysis, the average value of the maximum bending stress at the end of the pipe double-limb double-plate gusset plate is 64MPa. The maximum bending stress at the end of the pipe single-limb single-plate gusset plate is 340MPa, and its ratio relationship is 5.3 times. Thus, it is basically consistent with the conclusions of the previous theory.

#### 4.3.3. Comprehensive Analysis of Plate End Stress of PSLSPJ and PDLDPJ

**Figure 19:**
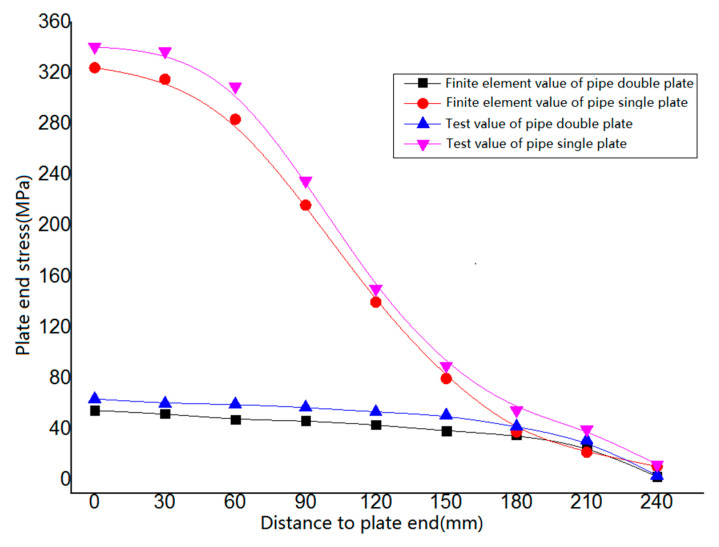
Comparison of bending stress test value and finite element value of plate end.

**Figure 20:**
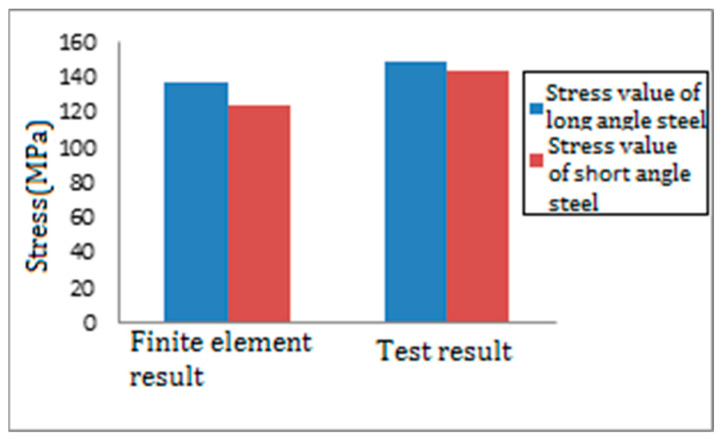
Comparison of stress test value and finite element value on long and short angle steel.

Analyze the bending stress in the dangerous area of the welding between the gusset plate and the leg member. Establish a path along the length direction of the welding between the gusset plate and the leg member, extract the bending stress along the width direction of the gusset plate. Analyze the plate end bending stress of the PSLSPJ and the PDLDPJ. The comparison results between the finite element value and the experimental value are shown in Figure 19. Extract the stress values on the long and short angle steel. The comparison results between the finite element values and the experimental values are shown in Figure 20.

It can be seen from Figure 19 that, in the finite element results, the maximum bending stress at the end of the gusset plate of the connection form of PSLSPJ is 324.20 MPa. The maximum bending stress at the end of the gusset plate is 59.90 MPa. The stress ratio at the end of the gusset plate is 5.41 times. The test results are basically consistent with the finite element results and theoretical values. The results show that, under the same external load, the bending stress at the end of the gusset plate can be greatly reduced.

It can be seen from Figure 20 that, in the finite element results, the stress on the long and short angle steel is found in Equation (20).

(20) $$\sigma_{\mathrm{long}}=137\;\mathrm{MPa}\;\;\sigma_{\mathrm{short}}=124\;\mathrm{MPa}$$

The stress ratio of long and short angle steel is 1.1:1. The comparison between the finite element results and the test results shows that the stress distribution on the long and short angle steel meets the ratio of 1:1. It is consistent with the assumption of equal force distribution on long and short angle steel in the theoretical analysis.

## 5. Conclusions

In this paper, a new type of joint connection, PDLDPJ, is proposed. The mechanical performance of PDLDPJ is systematically analyzed by using theoretical analysis, finite element analysis, and scale test methods. Compared with the traditional PSLSPJ, the following conclusions are obtained:

1. Based on the shortcomings of the tube single-leg single-plate joint, such as the eccentric stress state of the inclined angle steel and the large bending stress of the gusset plate, a new type of connection joint is proposed—the PDLDPJ. The PDLDPJ makes inclined angle steel in the axial tension and compression state and the gusset plate in the plane stress state, so as to improve the strength of the joint.
2. Under the same load, the relationship between the stress distribution of the angle steel section and the stress at the end of the gusset plate is compared and analyzed. The theoretical results are as follows:

On the one hand, through a theoretical derivation, the stress relationship of the angle steel section under eccentric load and axial load is obtained: The maximum stress of the angle steel section under eccentric load is 2.5 times of that under axial load.

On the other hand, the stress relationship between the gusset plate end of PSLSPJ and PDLDPJ under the uniform load satisfies Equation (11). Plate end stress is related to the plate thickness and angle steel thickness of the gusset plate. From the expression, it can be seen that the plate end stress of the PSLSPJ is at least four times that of the PDLDPJ. Therefore, the PDLDPJ can effectively reduce the plate end stress of the gusset plate.

3. Static load tests of PSLSPJ and PDLDPJ are completed. The test results show that the bearing capacity of PDLDPJ is higher than that of PSLSPJ. Plate end stress of PSLSPJ is 5.3 times that of PDLDPJ. The average stress ratio of long and short angle steel in PDLDPJ is 1:1.03. Under the same conditions, the finite element modeling analysis of the joint is performed. The finite element results are consistent with the experimental results and theoretical analysis results.

## Figures and Tables

**Table 1:** Dimensional specifications for finite element models of PSLSPJ and PDLDPJ.

Joint Name	PDLDPJ (mm)	PSLSPJ (mm)
Leg members	Φ140 × 8	Φ140 × 8
Diagonal members	L50 × 5	L50 × 5
Gusset plate	460.8 × 4	460.8 × 8
Bolt model	Φ8	Φ8
Weld size	460.8 × 6	460.8 × 6

**Table 2:** Test value and finite element value in the weld of PSLSPJ.

Measuring Point No.	Test Value (MPa)	Finite Element Value (MPa)	Measuring Point No.	Test Value (MPa)	Finite Element Value (MPa)
1	340.5	324.2	19	316.2	304.1
2	336.8	297.2	20	312.5	291.3
3	309.3	283.6	21	234.3	227.4
4	235.2	216.1	22	214.6	189.5
5	150.5	150.5	23	125.5	138.9
6	48.7	50.2	24	45.1	76.4
7	38.1	37.6	25	35.1	63.2
8	22.5	25.1	26	19.9	25.3
9	11.2	12.5	27	19.6	12.6

**Table 3:** Test value and finite element value in weld of PDLDPJ.

Measuring Point Number	Test Average Stress (MPa)	Finite Element Average Stress (MPa)	Measuring Point Number	Test Average Stress (MPa)	Finite Element Average Stress (MPa)
1	97	92.84	23	−32	−30.73
2	96	91.67	24	−24	−22.17
3	96	91.31	25	4	3.53
4	102	96.31	26	29	28.62

## Data Availability

The data presented in this study are available on request from the corresponding author.
